# Comparison of brush and biopsy sampling methods of the ileal pouch for assessment of mucosa-associated microbiota of human subjects

**DOI:** 10.1186/2049-2618-2-5

**Published:** 2014-02-14

**Authors:** Susan M Huse, Vincent B Young, Hilary G Morrison, Dionysios A Antonopoulos, John Kwon, Sushila Dalal, Rose Arrieta, Nathaniel A Hubert, Lici Shen, Joseph H Vineis, Jason C Koval, Mitchell L Sogin, Eugene B Chang, Laura E Raffals

**Affiliations:** 1 Department of Pathology and Laboratory Medicine, Brown University, Providence, RI, USA; 2 Department of Internal Medicine, Division of Infectious Diseases, Ann Arbor, MI, USA; 3 Department of Microbiology and Immunology, University of Michigan Medical School, Ann Arbor, MI, USA; 4 Josephine Bay Paul Center, Marine Biological Laboratory, Woods Hole, MA, USA; 5 Institute for Genomics and Systems Biology, Argonne National Laboratory, Argonne, IL, USA; 6 Department of Medicine, Section of Gastroenterology, The University of Chicago, Knapp Center for Biomedical Discovery, Chicago, IL, USA; 7 Department of Internal Medicine, Division of Gastroenterology and Hepatology, Mayo Clinic, Rochester, MN, USA

**Keywords:** Microbiome, Ulcerative colitis, Mucosal biopsy, Cytology brush, Microbial sampling, Mucosal brushing, Microbiome methods

## Abstract

**Background:**

Mucosal biopsy is the most common sampling technique used to assess microbial communities associated with the intestinal mucosa. Biopsies disrupt the epithelium and can be associated with complications such as bleeding. Biopsies sample a limited area of the mucosa, which can lead to potential sampling bias. In contrast to the mucosal biopsy, the mucosal brush technique is less invasive and provides greater mucosal coverage, and if it can provide equivalent microbial community data, it would be preferable to mucosal biopsies.

**Results:**

We compared microbial samples collected from the intestinal mucosa using either a cytology brush or mucosal biopsy forceps. We collected paired samples from patients with ulcerative colitis (UC) who had previously undergone colectomy and ileal pouch anal anastomosis (IPAA), and profiled the microbial communities of the samples by sequencing V4-V6 or V4-V5 16S rRNA-encoding gene amplicons. Comparisons of 177 taxa in 16 brush-biopsy sample pairs had a mean R^2^ of 0.94. We found no taxa that varied significantly between the brush and biopsy samples after adjusting for multiple comparisons (false discovery rate ≤0.05). We also tested the reproducibility of DNA amplification and sequencing in 25 replicate pairs and found negligible variation (mean R^2^ = 0.99). A qPCR analysis of the two methods showed that the relative yields of bacterial DNA to human DNA were several-fold higher in the brush samples than in the biopsies.

**Conclusions:**

Mucosal brushing is preferred to mucosal biopsy for sampling the epithelial-associated microbiota. Although both techniques provide similar assessments of the microbial community composition, the brush sampling method has relatively more bacterial to host DNA, covers a larger surface area, and is less traumatic to the epithelium than the mucosal biopsy.

## Background

In recent years, clinical research has highlighted the important role of commensal gut microbes in human health. The gut microbiota not only plays a major role in health, but also in the etiopathogenesis of complex immune disorders such as inflammatory bowel diseases, type I diabetes, metabolic disorders, irritable bowel syndrome, and cancer [1-8]. Most studies of human gut microbial communities rely on the non-invasive collection of stool samples. While this has proven informative, analyses of the fecal microbiota may miss opportunities to understand the role of the mucosa-associated microbes, which live in close proximity to the intestinal epithelium and may not be adequately represented in the luminal (fecal) samples. The mucosa-associated microbes may also play a more important role in diseases such as inflammatory bowel disease (IBD), where direct interactions likely occur between the host immune system and the microbes living in the mucus layer of the epithelium. The optimal approach for sampling the mucosa-associated microbiota has not yet been defined.

Studies of the mucosa-associated microbes in the gut typically make use of mucosal biopsies obtained during endoscopic procedures. While individual biopsies present minimal risk, repeated biopsies, particularly in an individual with underlying bowel inflammation, can lead to bleeding and infection. Additionally, mucosal biopsies include only a small surface area and can therefore lead to sampling bias, especially for rare taxa, if the bacterial populations have a patchy distribution. Mucosal biopsies frequently contain a large proportion of contaminating host DNA, which complicates metagenomic and other molecular analyses.

In contrast, mucosal brushing reduces the risks associated with mucosal biopsies and provides a more representative sampling of the mucosal surface. Mucosal brushings obtained during an endoscopic procedure are less invasive than mucosal biopsies. They have the advantage of covering a larger surface area of the bowel than a biopsy. Furthermore, brushings do not remove attached epithelium and subsequently should have a smaller proportion of host cells.

We prospectively followed patients with a history of ulcerative colitis (UC) who had undergone total proctocolectomy with ileal pouch anal anastomosis (IPAA). In an effort to identify an accurate but less invasive sampling method of the mucosal-associated microbiota, we sought to determine whether mucosal brushings are a comparable sampling method to the mucosal biopsy. To test whether the two techniques provide equivalent sampling of the mucosa-associated microbiota, we performed paired samplings using both brush and biopsy sampling in a subset of subjects of our larger IPAA patient cohort. To understand the variation due to sampling technique as opposed to variation inherent in the DNA amplification and sequencing, we also evaluated the reproducibility of technical replicates by independently reamplifying and resequencing 25 samples.

## Methods

### Sample collection

We collected 16 paired mucosal biopsies and mucosal brushings from four patients with UC enrolled in a longitudinal study that explores the role of the enteric microbiota in pouchitis. All patients underwent colectomy and IPAA as part of their treatment. Samples from the ileal pouch were collected prior to closure of the diverting ileostomy and at nine additional time points over the course of 2 years following reinstitution of the fecal stream as fully described by Young *et al*. [9]. These occurred at 14, 16, 20, and 24 months post-surgery for patient 200; 12, 16, and 17 months for patient 206; 8, 12, 16, 18, and 20 months for patient 207; and 4, 12, 17, and 20 months for patient 210. All patients gave written informed consent to participate in this research study, with the understanding that the results were to be published. The Institutional Review Board of the University of Chicago Medical Center approved the study protocol.

None of the patients underwent any form of bowel preparation or lavage, which can distort the luminal and mucosa-associated complement of microbiota. In all cases, the mucosal brushings were obtained prior to biopsies to prevent contamination of the brush samples with blood. Mucosal brushings were performed using Cook Medical Endoscopy Cytology Brushes (Cook Medical no.: G22108). The cytology brush was advanced through the colonoscope channel. Brushings were done using broad or long strokes, applying gentle pressure to the pouch mucosa. Sampling of a larger surface area of the ileal pouch epithelium was therefore possible with each brushing, and effort was made to sample areas of the ileal pouch with some stool adherent to the pouch wall to ensure that the passage of harder stool had not scraped away mucosa-adherent bacteria. The brush was then retracted into its sterile sheath prior to withdrawal through the colonoscope channel. Mucosal biopsies were taken using standard biopsy forceps. Samples were placed on dry ice and then stored at −80°C until processing.

### Amplicon library construction and sequencing

During the course of the research project, we shifted from using the Roche GS FLX Titanium sequencing platform (454) to the Illumina MiSeq platform. Because the MiSeq read length is shorter than that of the GS FLX, we targeted a smaller 16S region to optimize read quality. Samples from patient 207 at 20 months and patient 210 at 12, 17, and 20 months were sequenced using Illumina. We sequenced all other samples using the GS FLX. We have included both sets of data to demonstrate that the brush and biopsy results are similar with both technologies.

Amplicon libraries for GS FLX Titanium sequencing span the V4-V6 16S rRNA region (sequenced from the 1046R towards 518 F primer, ~546 nt). Illumina MiSeq amplicons span the V4-V5 16S rRNA region (paired-end sequenced between 518 F and 926R, ~408 nt). The 16S-specific primers and the sequencing adaptors are shown in Additional file 1: Table S1. Primers for GS FLX sequencing contain the GS FLX Titanium amplicon adaptors, a forward or reverse 16S-specific primer, and a 5-nt multiplexing identifier (MID) between the sequencing primer binding site and the 16S-specific region. Primers for Illumina sequencing contain the bridge adaptors necessary for clustering, sequencing primer binding sites, forward or reverse 16S-specific primer, and an in-line MID (forward primer) or index that is sequenced in a separate indexing read (reverse primer). We use a combination of in-line MID and index to multiplex libraries. The 16S-specific primers contain degenerate sites or, in the case of 926R, represent a combination of three distinct oligonucleotides in order to capture broad eubacterial diversity.

Our GS FLX amplification and sequencing protocols are described in Marteinsson et al. [10] and Young et al. [9]. The V4-V5 amplicons for Illumina sequencing were generated using a two-step amplification procedure. The first step reaction mix contained 1× Platinum HiFi Taq polymerase buffer, 2 mM MgSO_4_, 0.2 mM dNTPs, 0.4 μM of the forward and reverse 16S-only primers (Additional file 1: Table S1), 10–30 ng genomic DNA, and 10 units of Platinum HiFi Taq polymerase (Life Technologies, Carlsbad CA) in a volume of 100 μl. This mix was divided into three replicate reactions before cycling. Cycling conditions were: an initial 94°C, 3-min denaturation step; 25 cycles of 94°C for 30 s, 60°C for 45 s, and 72°C for 60 s; and a final 2-min extension at 72°C. The triplicate PCR reactions were pooled after amplification and purified using Ampure XP (Beckman Coulter, Indianapolis IN) in a 1:1 volume ratio according to the manufacturer’s protocol. The purified PCR products were eluted in 20 μl of Qiagen buffer EB (Qiagen, Valencia CA); 4 μl of the eluate served as template for the second step. Reaction components were the same as in the first step except that the amount of Taq was reduced to 2 units and a reaction volume of 25 μl was used. The entire 25 μl volume was amplified as above for five cycles. PCR products were size-selected using a 1:1 ratio of Ampure XP to sample to exclude primer-dimer products. Libraries were quantitated using PicoGreen QuantIt (Life Technologies, Carlsbad CA) and pooled in equimolar amounts. The final pool was quantitated using the KAPA library quantification protocol (Kapa Biosystems, Boston MA). Libraries were sequenced on an Illumina Miseq 250-cycle paired-end run. The combination of CASAVA 1.8.2 to identify reads by index and a custom Python script that resolved barcodes demultiplexed the data sets.

Technical replicate pairs were sequenced using the Roche GS-FLX Titanium protocol as described above. We tested the reproducibility of our amplification and sequencing early in the experiment, and the samples are from patient 200 (initial visit, 2, 4, and 8 weeks), patient 206 (initial visit, 3, 5, and 8 weeks), patient 207 (initial visit, 2, 4, and 8 weeks), and patient 210 (initial visit, 3 and 5 weeks). All of the samples from patients 206 and 207 and the first two samples from patient 210 were duplicated biopsies (sampled twice on the same visit), and we used both in the technical replication experiment, for a total of 25 technical replicate pairs (4 from patient 200, 8 from patient 206, 8 from patient 207, and 5 from patient 210).

### qPCR of bacterial and human DNA

Quantitative PCR (qPCR) was used to assay the quantity of bacterial rRNA operons in the samples normalized to a single-copy host gene (TNF-α). Real-time qPCR was performed on a Roche Lightcycler 480 using LightCycler 480 96-well plates (Roche) covered with LightCycler 480 sealing foil (Roche). Each 20-μl reaction was performed in triplicate and contained 10 μl 2× SYBR Green PCR Master Mix (Roche), 1 μl of each primer (10 μM concentration, 0.5 μM final), 1 μl template DNA, and 7 μl of Hypure Molecular Biology grade water (Thermo Scientific). For detection of the bacterial signal, we used the primer-probe combination of: forward primer (5’-TCCTACGGGAGGCAGCAGT-3’), reverse primer (5’-GGACTACCAGGGTATCTAATCCTGTT-3’), and probe (5’-[6-carboxyfluorescein]-CGTATTACCGCGGCTGCTGGCAC-[6-carboxytetramethylrhodamine]-3’) [11]. The amplification reaction conditions were 95°C for 10 min and 40 cycles of 95°C for 15 s and 60°C for 1 min. Detection of the host signal used a primer-probe combination of forward primer (TNFa_hu_se; 5’- AGGAACAGCACAGGCCTTAGTG-3’), reverse primer (TNFa_hu_as; 5’- AAGACCCCTCCCAGATAGATGG-3’), and probe (TNFa_hu_probe; 5_-Cy5-CCAGGATGTGGAGAGTGAACCGACATG-Iowa Black RQ-3_) [12]. Amplification of the host signal began with incubation at 95°C for 10 min, followed by 45 cycles of 95°C for 20 s and 64°C for 30 s. All oligonucleotides used were ordered from Integrated DNA Technologies.

Amplification product size was verified using agarose gel electrophoresis, and quantification of product copy numbers was inferred from a dilution series of purified plasmids containing a representative target (*Escherichia coli* for the bacterial 16S rRNA encoding gene and human TNFα). Templates for these positive controls were amplified from *E. coli* TOP10 using 8F and 1492R primers [13] and human genomic DNA using TNFα_hu_se and TNFα_hu_as primers [12], respectively. Amplicons were then cloned using the TOPO TA cloning kit (pCR4-TOPO vector; Invitrogen) and sequences verified using Sanger sequencing. Purified plasmids were then quantified using the Qubit (Invitrogen), and standards ranging in concentration from 10^9^–10^1^ plasmid copies/μl were prepared. These standards were run in parallel with our samples, and a standard curve was generated to assess target copies within each sample.

### Data analysis

Quality filtering for pyrosequencing data retained reads that (1) had exact matches to the MID and proximal primer (1046R), (2) contained no ambiguous bases (Ns), and (3) had average quality scores greater than 30 [14]. Because the length of the amplified regions exceeded the high-quality read length capability of the GS FLX, our informatics pipeline truncated all reads at a known conserved motif within the amplicon between the V4 and V5 regions, TGGGCGTAAAG, or discarded reads that lacked the anchor sequence. The Illumina MiSeq reads were merged and quality filtered using custom Python scripts that allowed no more than three mismatches in the overlap region of paired end reads. This process is a modification of the methods described in Eren et al. [15] (code available at https://github.com/meren/illumina-utils). UCHIME [16] removed chimeras using both the reference mode against Gold reference set [17] and the *de novo* mode. We assigned taxonomy using GAST [18] to compare reads against the SILVA 16S reference database [19] including, whenever possible, assignments to the genus level. We uploaded the trimmed and quality-filtered sequences to the Visualization and Analysis of Microbial Population Structures website (VAMPS) (http://vamps.mbl.edu) [20] where they are publicly available under projects VBY_BRBI_Bv6v4 and VBY_BRBI_Bv4v5. Simple relative abundance, mean, standard deviation, and Chao alpha diversity [21] calculations were performed in R [22,23]. Alpha diversity was calculated after subsampling all data sets to the minimum number of reads (2,023), so that estimates could be compared.

We define technical replicates as two independent PCR amplifications and subsequent sequencing from the same DNA extraction of a specific sample. We define brush-biopsy pairs as a biopsy sample and a brush sample taken from the ileal pouch of the same patient at the same visit.

To assess the potential effect of sampling bias on detection of taxa and determination of relative abundances using the brush and biopsy techniques, we performed both the paired Student’s *t*-test (parametric) and Wilcoxon rank sum test (non-parametric) in R (t.test and wilcox.test, paired = TRUE for both) comparing the brush data sets to the biopsy data sets. Although non-parametric tests are often considered more robust, microbial communities typically contain a long tail of low abundance taxa, and small variations in abundances can cause a large shift in rank, possibly leading to incorrect estimates of the number of taxa that differ significantly between data sets. We corrected for multiple experiments (false discovery rate or *q*-value) using the Benjamini-Hochberg algorithm (p.adjust in R). We considered taxonomic differences to be statistically significant if their *q*-values were less than or equal to 0.05.

As an additional check on differences between the brush and biopsy techniques, we also used the LDA Effect Size method (LEfSe) [24]. LEfSe is a microbial community biomarker tool that identifies features (taxa) that differ between classes of samples (brush vs. biopsy). LEfSe uses the non-parametric Kruskal-Wallis rank-sum test. While we believe that statistics explicitly assessing sample pairs are more sensitive to changes within pairs when using diverse samples such as stool, LEfSe is used frequently in microbial analyses. To test whether the selection of sampling method affects common downstream analyses, we included an LEfSe analysis. We uploaded the abundance matrix of all taxa with a mean count greater than five reads. We used per-sample normalization, default values of alpha (= 0.05) and the threshold log LDA score (= 2). We ran the analysis using both stringency levels (“all against all” and “one against all”).

To assess overall pairwise similarity, we compared the number of sequences in one sample assigned to each taxon with the number of sequences assigned to the same taxon in the other sample. We made this comparison using the Pearson product–moment correlation coefficient (R^2^) between the two samples of each pair for all pair sets (technical replicates and brush-biopsy pairs) using the cor function in R [22,23]. Taxa present in one data set of a pair and not in the other are represented in the latter by an abundance value of 0. Taxa not present in either sample are not included in the analysis. We subsampled (randomly, without replacement) the larger data set in each pair to match the number of reads in the smaller data set using a custom perl script (random_mtx) so that each pair represented two data sets with the same number of reads. Unequal sampling depth biases the Pearson correlation because of the presence of many low abundance taxa detected in the larger data set but not detected in the smaller one. We prefer Pearson’s correlation to Spearman’s for stringency, because it tests not simply whether the rank order of taxa abundances is similar (Spearman), but also that the relative abundance values (Pearson) of taxa data sets are similar. Most microbial community analyses are based on the relative abundances of taxa, not their rank.

We carried out a principal coordinates analysis (PCoA) and generated a UPGMA dendrogram for the brush-biopsy pairs based on the Morisita-Horn distance metric using VAMPS to demonstrate how the brush and biopsy pairs cluster in the larger set of samples. Both of these clustering methods are commonly used for microbiome analyses as reported in the current literature. We used the complete data sets for community distance comparisons because the Morisita-Horn community distance metric compensates for differences in sampling depth, precluding the need to randomly subsample sequences to the same depth.

The relative proportion of bacterial to human DNA was calculated by first generating a standard curve relative to the dilution series performed during qPCR in order to correlate fluorescence with copy number. The equation for the standard curve was then applied to the signals detected for each target and used to calculate copy numbers of the target (16S rRNA encoding gene or *TNF*-α encoding gene) per reaction and per ng of template. The ratio of 16S rRNA-based copy number relative to *TNF*-α copy number per ng of DNA was then used to infer gross fold differences between brush and biopsy samples for each of the samples. These values were then averaged by patient so that the standard deviations could be calculated.

## Results and discussion

### Overview of the pouch microbiota

Following reinstitution of the fecal stream through the ileal pouch, there is an evolution of the microbiota as the community begins to take on features more similar to those of the healthy colonic microbial community [9]. In the present study, we sampled the pouch microbiota at different time points at various states of health and disease (inflammation of the pouch). The ten most abundant taxa across all samples include (in order): *Bacteroides*, *Lachnospiraceae* (genus unknown), *Clostridium*, *Enterobacteriaceae* (genus unknown), *Blautia*, *Roseburia*, *Epulopiscium*, *Peptostreptococcaceae* (genus unknown), *Acidaminococcus* and *Streptococcus*. Together, the six most abundant taxa represent an average of 70% (± 10%) of taxa across the samples, and the top ten together represent 82 ± 3.5%. The variation of microbial composition is quite large across the samples, with *Bacteroides*, the most abundant taxon, ranging from 0 to 60% (25 ± 23%) and *Lachnospiraceae* ranging from 9.5%-34% (20 ± 8%). Intriguingly, although the standard deviation of the abundance of the top ten taxa ranges from 3.2 to 23, the coefficients of variation (standard deviation/mean) are all equal to 16, implying a similar relative variation for all of these taxa. The Chao alpha diversity estimates for these samples averaged 39 (range: 25–96), but included a very broad range of confidence limits from 22 to 410, implying that the minimum sample size of 2,023 is not adequately characterizing many of these communities. Fortunately, all but 3 of the 32 data sets had more than 5,000 high-quality reads.

### Taxon-specific statistical analyses

None of the taxa had a false discovery rate of *q* ≤ 0.05 with either Student’s *t*-test or the Wilcoxon rank sum test; hence, no taxa exhibit statistically significant variation between the two methods. We used LEfSe to confirm our results. LEfSe identified no discriminative taxa between brush and biopsy samples.

### Correlation analysis for sample pairs

**Figure 1:**
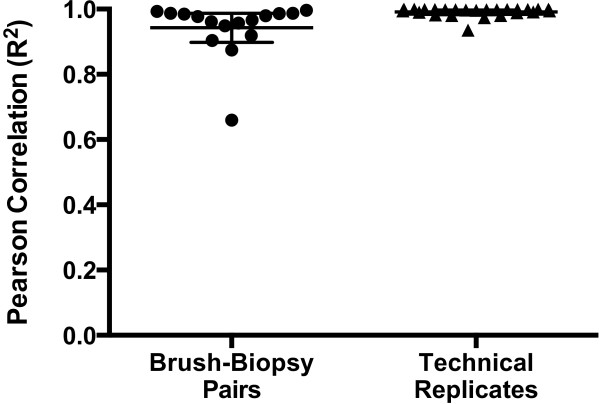
**Correlation of sample pairs.** Pearson product–moment correlation values for two sets of paired samples: brush vs. biopsy method sample pairs and paired technical replicates of the DNA amplification and sequencing process. Excepting one outlier, brush-biopsy pairs correlated exclusively above 0.87, with a mean R^2^ of 0.94. All technical replicate pairs correlated above 0.93 with a mean R^2^ of 0.99.

The 16 brush-biopsy pairs (samples using both techniques from the same patient on the same visit) had a mean genus-level Pearson R^2^ of 0.94 ± 0.04, with all but two sample pairs having an R^2^ greater than 0.90 (Figure 1). This demonstrates that the taxon-by-taxon abundances are highly reproducible between the two methods. The 25 technical replicate pairs (same DNA extraction, but independent PCR amplification and sequencing) had a mean Pearson R^2^ of 0.99 ± 0.01, with all pairs having an R^2^ > 0.93, demonstrating excellent reproducibility of the rRNA gene amplification and of the DNA sequencing across the Roche GS FLX Titanium and Illumina MiSeq platforms. Sample 206–16 months is an outlier in our analysis (R^2^ = 0.66). There could be many reasons for this, including heterogeneity of the pouch, imperfect sample collection, or minor contamination of the sample during processing. Unfortunately, we did not have sufficient DNA remaining to repeat the amplification and sequencing and verify the result.

### Community distance metrics

**Figure 2:**
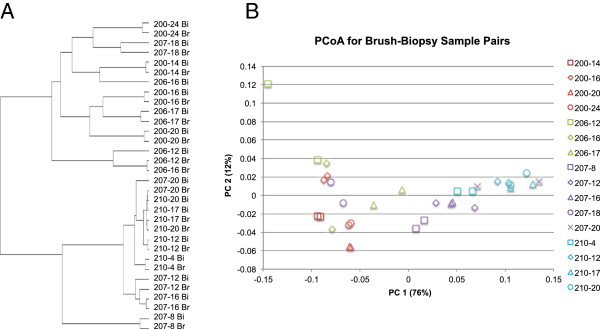
**Clustering of sample pairs.** UPGMA dendrogram **(A)** and principle coordinate analysis (PCoA) **(B)** of the brush-biopsy sampling pairs. Results show the close clustering of samples from the same brush-biopsy pairs, as evidenced by the short branch lengths in the dendrograms and the close proximity of the points in the PCoA.

The UPGMA clustering dendrogram analysis of the brush-biopsy pairs shows that 3 of the 16 pairs (206–16 months, 207–20 months, and 210–20 months) did not form a clade together as would be expected. This would suggest differences between sampling methods in these samples. Based on our taxonomic evaluation, however, this does not represent a consistent bias between the two methods. If these three pairs were excluded, all other sample pairs did appear in the same clade (Figure 2A). The dendrogram contains many short inter-pair branch lengths for patients 207 and 210, implying that pouch microbiota at these patient visits were very similar.

We illustrate these same results in a PCoA plot (Figure 2B). The average pairwise distance of brush-biopsy pairs is 0.07 ± 0.09, including the outlier 206–16 (distance = 0.34). The samples of pair 206–12 appear different, but their pairwise distance is only 0.16. While several of the samples do not appear to cluster exclusively by pair, these data represent multiple visits from only four patients who have recently undergone a similar major surgery, and their pouch microbiome is quite consistent across many of the visits. In other words, the samples are so similar that despite the reproducibility of the sampling methods, multiple pairs cluster together. Overall, the biopsy and brush methods fall into the same group of samples. Dendrogram and PCoA plots do not provide information about specific taxa that might differ across the sampling methods, but they are commonly used in microbiome analyses. Our combined results demonstrate that brush and biopsy sampling methods provide similar sample clustering results.

### Bacterial DNA yields

**Figure 3:**
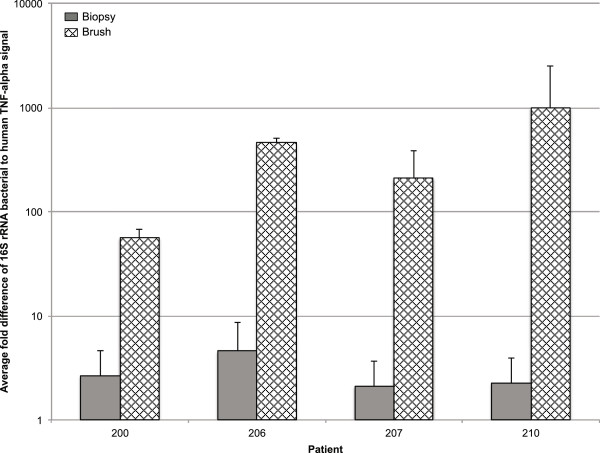
**Comparison of DNA yields.** Comparison of bacterial and human host DNA yields from brush and biopsy methods. The *bars* represent the averaged brush and the averaged biopsy samples for each patient. Brush samples show a much larger ratio of bacterial 16S DNA to human TNF-α.

While the taxa represented by the two sampling methods are similar, brush sampling yields an improved ratio of bacterial DNA to human DNA (Figure 3). The average fold difference between brush and biopsy samples for the bacterial signal (normalized to human) was 21.2-fold higher for patient 200 and even greater for the other three patients: 100.7-fold for patient 206, 99.7-fold for patient 207, and 441.3-fold for patient 210. The large variation in ratio of bacterial to human DNA is likely caused by a variation in the fecal matter, which can sometimes be very watery and thin, dominated by mucus, or can be more characteristic of a full stool sample with a large bacterial load.

Nonetheless, the favorable bacterial to human DNA ratio is a boon for metagenomic and other analyses that require separating host from microbiome sequences and for low biomass samples where DNA extraction and amplification have very low yields.

## Conclusions

These data show that mucosal brushings and mucosal biopsies provide comparable results for sampling the mucosa-associated microbiota in the pouch of UC patients. Paired samples showed a 0.94 Pearson product–moment correlation (R^2^). We detected no statistically significant taxonomic differences attributable to the sampling method. The impact of the sampling technique on the patient, however, is quite different. Repeated biopsies traumatize the epithelium, whereas mucosal brushings are less invasive and carry minimal to no risk, an attractive feature to both institutional review boards and patients. Brush samples also offer clear advantages in providing a much higher ratio of bacterial DNA to host DNA and sampling from a much larger surface area, which should provide better representation of rare taxa and of a heterogeneous mucosal layer, as can occur in IBD patients.

Our study also demonstrates that technical replicates (independent rRNA gene amplification and sequencing from the same DNA extraction) correlate nearly perfectly (R^2^ = 0.99).

Our samples derive from the small bowel epithelium of the ileal pouch. While most samples for microbial analysis come from the colon, our prior work has shown that the microbial community of the pouch in healthy individuals is similar to that of the healthy colon [9]. We believe our findings will be applicable to studies of the small bowel and the colon, but additional research is required to verify this.

Although great progress has been made in understanding the role of the microbiota in health and disease, longitudinal studies with repeated sampling of microbial communities over time increase the ability to identify potential causal relationships. Targeted sampling of the mucosa-associated microbiota has relied primarily on biopsies taken during endoscopy. Looking forward, our results may help with the innovation of alternative sampling methods of the mucosa-associated microbiome. Repeated endoscopy is cost prohibitive and invasive. An anoscope with a cytology brush, on the other hand, can easily sample the mucosa-associated microbiome during an office visit. As was the case with our flexible sigmoidoscopic exams, there would be no need for bowel preparation, which can distort the natural complement of mucosa-associated microbiota.

## Availability of supporting data

The trimmed and quality-filtered sequences have been uploaded to the Visualization and Analysis of Microbial Population Structures website (VAMPS) (http://vamps.mbl.edu) [20] where they are publicly available under project names VBY_BRBI_Bv6v4 and VBY_BRBI_Bv4v5. Raw sequences are also available through the NCBI, BioProject ID PRJNA46315. Note that the sample names included in this publication have been modified to reflect the number of months the patients has been included in the study. The archived sample names are based on visit number. The sample numbers for patient 200, months 14, 16, 20, 24 are 200_14, 200_8, 200_9, 200_10. Sample numbers for patient 206 months 12, 16, 17 are 206_7, 206_8, 206_12. Sample numbers for patient 207 months 8, 12, 16, 18, 20 are 207_6, 207_7, 207_8, 207_12, 207_9. Sample numbers for patient 210, months 4, 12, 17, and 20 are 210_5, 210_7, 210_8, and 210_9. The letters “E” and “F” designate biopsy samples, and “GG” designates brush samples.

## Abbreviations

IBD: Inflammatory bowel disease; IPAA: Ileal pouch anal anastomosis; PCoA: Principle coordinate analysis; UC: Ulcerative colitis; UPGMA: Unweighted pair group method with arithmetic mean; VAMPS: Visualization and analysis of microbial population structures website.

## Competing interests

The authors declare that they have no competing interests.

## Authors’ contributions

VBY, EBC, LER, and MLS designed the experiment. LER and JK sampled the ileal pouch. JK, RA, SD, and LS were involved in patient recruitment and enrollment. DAA, NAH, JK, RA, and SD helped process samples and/or extract the DNA. DAA and JCK performed and analyzed the qPCR. JHV sequenced the DNA. HGM designed the primers. SMH analyzed the results. SMH, VBY, DAA, EBC, HGM, and LER wrote the manuscript. All authors read and approved the final manuscript.

## Supplementary Material

Additional file 1: Table S1 Amplification primer sequences for both the Roche GS FLX Titanium and Illumina MiSeq platforms and for amplification of both the V4-V6 (454) and V4-V5 (MiSeq) regions of the SSU rRNA (16S) gene. **Table S2.** Sequencing read counts for all brush-biopsy sample pairs including the total number of reads, the number of reads identified as low quality, the number of reads identified as chimeras, and the total number of remaining high-quality sequencing reads used for analysis. Click here for file
