# Supplementary material for: Comparison of brush and biopsy sampling methods of the ileal pouch for assessment of mucosa-associated microbiota of human subjects
Source: Microbiome. 2014 Feb 14;2:5. doi: 10.1186/2049-2618-2-5 (PMC3931571; doi:10.1186/2049-2618-2-5)
Supplement: Additional file 1: Table S1 — Amplification primer sequences for both the Roche GS FLX Titanium and Illumina MiSeq platforms and for amplification of both the V4-V6 (454) and V4-V5 (MiSeq) regions of the SSU rRNA (16S) gene. Table S2. Sequencing read counts for all brush-biopsy sample pairs including the total number of reads, the number of reads identified as low quality, the number of reads identified as chimeras, and the total number of remaining high-quality sequencing reads used for analysis. [file 2049-2618-2-5-S1.docx]

**Supplemental Table 1**

| **Primer** | **Sequence** | **Type** |
| --- | --- | --- |
| 454-A | CGTATCGCCTCCCTCGCGCCATCAG | Adapter |
| 454-B | CTATGCGCCTTGCCAGCCCGCTCAG | Adapter |
| Illumina R1 | AATGATACGGCGACCACCGAGATC- TACACTCTTTCCCTACACGACGCT- CTTCCGATCT | Bridge/ Sequencing adapter |
| Illumina R2 | CAAGCAGAAGACGGCATACGAGAT-xxxxxxGTGACTGGAGTTCAGACG- TGTGCTCTTCCGATCT | Bridge / Sequencing adapter, 6nt index |
| 518F | CCAGCAGCYGCGGTAAN | v4-v6 forward primer |
| 565F-a | TGGGCGTAAAG | v4-v6 Informatics landmark (anchor) |
| 1064R | CGACRRCCATGCANCACCT | v4-v6 reverse primer |
| 518F | CCAGCAGCYGCGGTAAN | v4-v5 forward primer |
| 926R1 | CCGTCAATTCNTTTRAGT | v4-v5 reverse primer |
| 926R3 | CCGTCAATTTCTTTGAGT | v4-v5 reverse primer |
| 926R4 | CCGTCTATTCCTTTGANT | v4-v5 reverse primer |

**Supplemental Table 2**

| **Sample** | **16S Region** | **Total Raw Reads** | **Low Quality Reads** | **Chimeric Reads** | **High-Quality Reads** |
| --- | --- | --- | --- | --- | --- |
| 200-14 | V4-V6 | 18,655 | 5,509 | 649 | 12,497 |
| 200-14 | V4-V6 | 35,671 | 8,279 | 7,512 | 19,880 |
| 200-16 | V4-V6 | 5,046 | 2,948 | 38 | 2,060 |
| 200-16 | V4-V6 | 37,454 | 9,435 | 7,970 | 20,049 |
| 200-20 | V4-V6 | 26,147 | 6,000 | 708 | 19,439 |
| 200-20 | V4-V6 | 40,508 | 10,027 | 10,560 | 19,921 |
| 200-24 | V4-V6 | 23,299 | 5,454 | 1,442 | 16,403 |
| 200-24 | V4-V6 | 41,348 | 10,244 | 10,885 | 20,219 |
| 206-12 | V4-V6 | 11,748 | 2,994 | 55 | 8,699 |
| 206-12 | V4-V6 | 37,212 | 8,687 | 10,307 | 18,218 |
| 206-16 | V4-V6 | 10,068 | 4,223 | 622 | 5,223 |
| 206-16 | V4-V6 | 37,824 | 8,962 | 8,667 | 20,195 |
| 206-17 | V4-V6 | 27,960 | 7,198 | 3,014 | 17,748 |
| 206-17 | V4-V6 | 33,812 | 9,229 | 8,608 | 15,975 |
| 207-8 | V4-V6 | 7,647 | 2,217 | 460 | 4,970 |
| 207-8 | V4-V6 | 11,270 | 2,865 | 3,244 | 5,161 |
| 207-12 | V4-V6 | 6,507 | 2,372 | 525 | 3,610 |
| 207-12 | V4-V6 | 29,693 | 11,918 | 6,959 | 10,816 |
| 207-16 | V4-V6 | 15,525 | 7,326 | 347 | 7,852 |
| 207-16 | V4-V6 | 29,102 | 11,726 | 3,207 | 14,169 |
| 207-18 | V4-V6 | 14,581 | 5,447 | 318 | 8,816 |
| 207-18 | V4-V6 | 44,361 | 10,332 | 15,304 | 18,725 |
| 207-20 | V4-V5 | 45,645 | 4,418 | 4,616 | 36,611 |
| 207-20 | V4-V5 | 33,827 | 2,688 | 3,697 | 27,442 |
| 210-4 | V4-V6 | 19,844 | 8,029 | 484 | 11,331 |
| 210-4 | V4-V6 | 31,760 | 11,731 | 9,088 | 10,941 |
| 210-12 | V4-V5 | 38,437 | 3,807 | 3,672 | 30,958 |
| 210-12 | V4-V5 | 48,551 | 4,472 | 5,157 | 38,922 |
| 210-17 | V4-V5 | 130,081 | 11,040 | 12,387 | 106,654 |
| 210-17 | V4-V5 | 27,167 | 2,138 | 3,277 | 21,752 |
| 210-20 | V4-V5 | 27,346 | 2,073 | 2,583 | 22,690 |
| 210-20 | V4-V5 | 7,719 | 887 | 802 | 6,030 |
